# Laparoscopic splenectomy in patients of β thalassemia: Our experience

**DOI:** 10.4103/0972-9941.68583

**Published:** 2010

**Authors:** Nirmal M Patle, Om Tantia, Prakash Kumar Sasmal, Shashi Khanna, Bimalendu Sen

**Affiliations:** Department of Minimal Access Surgery, ILS Hospital, Kolkata, India

**Keywords:** Laparoscopic splenectomy, pfannenstiel incision, thalassemia

## Abstract

**BACKGROUND:**

Laparoscopic splenectomy has become a standard treatment of various haematological disorders, but its feasibility in the setting of β thalassemia has not been established.

**MATERIALS AND METHODS:**

Fifty patients of β thalassemia underwent laparoscopic splenectomy between January 2006 and December 2008. “Anterior approach” method was practiced in all cases, with early ligation of splenic artery and delayed ligation of splenic vein. Specimen was extracted piecemeal via the umbilical port in initial 12 cases, while in 37 cases the specimen was extracted through a 7-8-cm pfannenstiel incision. Twelve patients of β thalassemia having grade IV splenomegaly with hepatomegaly were electively operated by conventional open method.

**RESULTS:**

The procedure was completed in 49 patients. One (2%) patient required conversion to open surgery. Mean operating time in the first 12 cases was 151 minutes (110-210 minutes), while in 37 cases of splenectomy completed laparoscopically it was 124 minutes (80-190 minutes) [*P* < 0.05]. Mean intra-operative blood loss was 73.8 ml (30–520 ml). No major intra-operative complications occurred. No patient required per-operative blood transfusion. Mean postoperative hospital stay was 4.7 days (2-11 days). Mean preoperative blood transfusion requirement was 11.98 units per patient per year, while mean postoperative blood transfusion requirement was 4.04 units [*P*< 0.05].

**CONCLUSION:**

Laparoscopic splenectomy is feasible and safe even in patients of β thalassemia with massive splenomegaly. Removal of specimen via a pfannenstiel incision significantly saves time, carries low morbidity and is a cosmetically acceptable alternative.

## INTRODUCTION

Splenectomy is a well-established therapeutic modality for the treatment of various types of haemolytic anaemias. It was first described for the treatment of hereditary spherocytosis in 1910.[1] β thalassemia is widely prevalent in the Indian population, and patients with this disease eventually require splenectomy for the treatment of hypersplenism. Over a course of time, experience and dexterity of surgeons in laparoscopy have grown. Technology in the field of imaging and in functional designing and construction of laparoscopic instruments has rapidly progressed. These facts, combined with enthusiastic acceptance of laparoscopic surgery by people, have caused a tremendous increase in the number and variety of surgeries performed laparoscopically.[2]

Laparoscopic splenectomy was first described by Delaitre in 1992.[3] Since then, laparoscopic splenectomy has become the procedure of choice in patients with normal-sized spleens who require splenectomy.[4,5] However, its application in the presence of significant splenomegaly has been controversial, with conversion rates ranging from 17% to 37%.[2,6–8] This becomes especially important in patients of β thalassemia, who usually have massive splenomegaly, thereby causing difficulty in intra-operative manipulation of the organ. Hence very large splenic size was considered a contraindication for laparoscopic splenectomy.[9,10] However, with growing expertise of surgeons and better instrumentation, even large-sized spleens are being removed laparoscopically.

We share our experience of laparoscopic splenectomy performed by a single surgical team in 50 patients of β thalassemia, from January 2006 to December 2008. The aim of this study was to evaluate the feasibility and safety of laparoscopic splenectomy in the difficult setting of β thalassemia. The large number of patients included in the study was due to the fact that the hospital runs a charitable trust for patients of thalassemia and all the patients of thalassemia are treated and operated free of cost.

## MATERIALS AND METHODS

Fifty patients of β thalassemia underwent laparoscopic splenectomy in the Department of Minimal Access Surgery, ILS Hospital, Kolkata, from January 2006 to December 2008. During the same period, 12 patients of β thalassemia with grade IV splenomegaly and hepatomegaly were electively subjected to open splenectomy. All the patients were operated by a single team headed by the second author. Indications of splenectomy included increased blood transfusion requirement, hypersplenism, haemosiderosis and abdominal pain or discomfort due to splenomegaly.

The patient data maintained on Microsoft Excel sheet were retrieved and analysed in terms of blood loss, operating time, conversion rates, complications and decrease in blood transfusion requirement postoperatively. Statistical analysis was done by using *t* test, and a *P* value of < 0.05 was considered significant. The patients were optimally prepared for surgery by preoperative blood transfusion to achieve Hb% of >10 gm%. All patients were immunized with hepatitis B, polyvalent pneumococcal and H. influenzae B vaccines 3 weeks prior to surgery.

**Figure 1:**
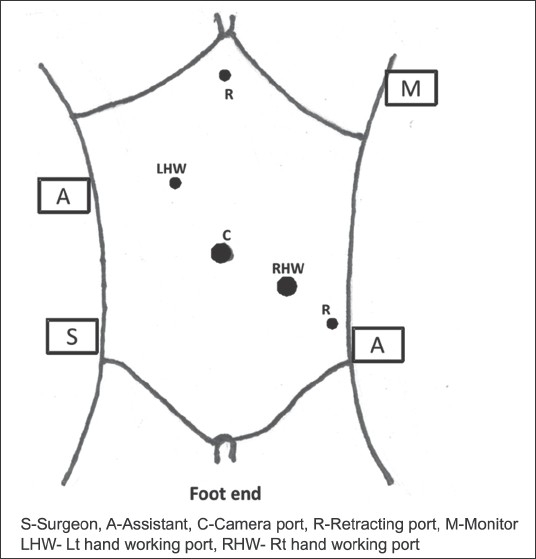
Port position and theatre layout

All patients were operated under general anaesthesia. A nasogastric tube and urinary catheter were introduced after induction of general anaesthesia. Patients undergoing laparoscopic splenectomy were placed in right semi-lateral position with the help of bolsters and 30° right lateral table tilt and reverse Trendelenberg position. The surgeon and the camera assistant stood on the right side of the patient; and the second on the left side of the patient. The monitor was placed at the left shoulder. A 10-mm optical port was placed at the umbilicus or at infra-umbilical or right paraumbilical level depending on the size of the spleen. A 10-mm right-handed working port was inserted in left mid-clavicular line just below the inferior border of the spleen. In patients with massive splenomegaly with spleen reaching up to the umbilicus, this port was placed last after retracting the spleen. A 5-mm left-handed working port was placed in transpyloric plane a little right of the midline. A 5-mm sub-xiphoid port was made for liver retraction. Another 5-mm retracting port was made in left anterior axillary line [Figure 1].

**Figure 2:**
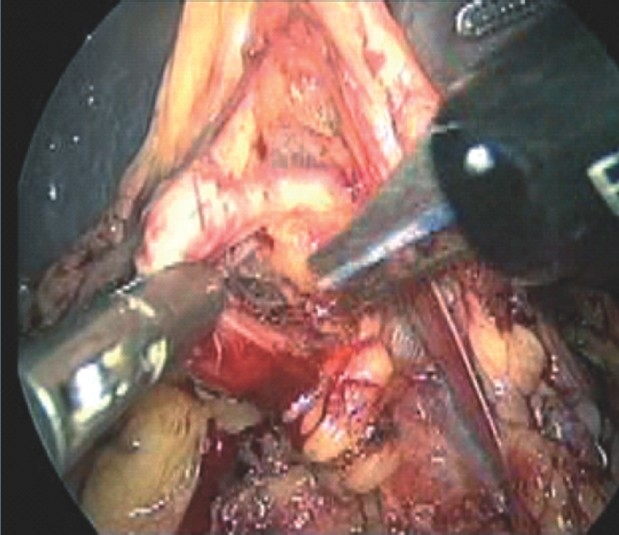
Dissection of splenic artery at splenic hilum

**Figure 3:**
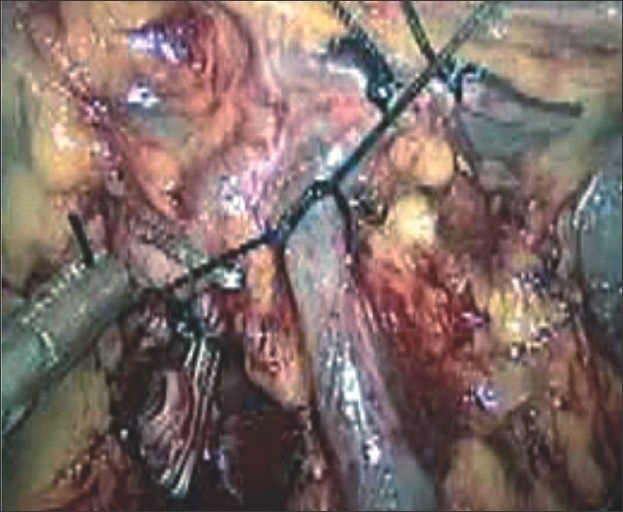
Ligation of splenic vein using mersilk 1 – 0 sutures

The peritoneal cavity was inspected for presence of accessory spleen. The first step was to mobilize the inferior pole of the spleen with the help of ultracision. Once the lower pole was free, the degree of mobility of spleen was increased, thereby facilitating the movement of the right-handed working instrument. The next step involved opening the lesser sac and ligating the short gastric vessels. We practiced the “anterior approach” method, dissecting the splenic artery above the superior pancreatic border at the hilum [Figure 2] and ligating it with 1–0 mersilk double ligatures in continuity [Figure 2]. The ligation of splenic vein was purposefully deferred till near-complete mobilization of the spleen was achieved. This involved dividing the splenocolic and lino-renal ligaments. Splenophrenic ligament was divided after ligation of the splenic vein. The splenic vein was doubly ligated with mersilk 1‐0 sutures [Figure 3]. Few cases required independent ligation of branches of the splenic artery and tributaries of the splenic vein. In the initial 12 cases, the spleen was retrieved by putting it in a lap bag and removed piecemeal after enlarging the umbilical port to a size of 4‐5 cm. It required a lot of time and patience. Hence later on, in 37 cases, where the procedure could be completed laparoscopically, a 7‐8–cm Pfannenstiel incision was used for removal of the specimen intact. The lower pole was first pulled out of the incision. Then along with good muscle relaxation, external abdominal wall pressure was applied, which aided removal of the organ. Abdominal drain was kept in the splenic bed.

Postoperative care involved injectable antibiotics (second-generation cephalosporin) for 48 hours and intravenous analgesics. The drain was removed 24-48 hours after surgery. Liquid diet was initiated 24 hours postoperatively. Early mobilization and chest physiotherapy were encouraged. The patients were discharged once they tolerated normal diet and were off injectable analgesics.

## RESULTS

Between January 2006 and December 2008, 50 patients of β thalassemia underwent laparoscopic splenectomy, and 12 patients underwent elective conventional open splenectomy. Among 50 patients of laparoscopic splenectomy, there were 29 (58%) males and 21 (42%) females. The median age of the patients was 15.7 years (range, 4-31 years). The procedure of laparoscopic splenectomy could be completed in 49 patients, and 1 (2%) patient required conversion to open method due to per-operative bleeding and perisplenic adhesions. With growing experience and familiarity with the procedure, the threshold for subjecting the patient to elective open procedure became higher. Accessory spleens were found in 6 (12%) patients. One patient was diagnosed to have retained accessory spleen by ultrasonography at 9 months’ follow-up. This was removed laparoscopically after its detection. Excision of this accessory spleen by re-laparoscopy has not been included in the current series. Mean craniocaudal splenic diameter as measured on ultrasound was 18.4 cm (range, 12-28 cm). The mean intra-operative blood loss was 73.8 ml (range, 30-520 ml). The mean operating time in the initial 12 cases where the spleen was removed after putting it into a lap bag was 151 minutes (range, 110-210 minutes). The mean operating time in 37 cases where a pfannenstiel incision was used for specimen removal was 124 minutes (range, 80-190 minutes) (*P*< 0.05). None of the patients required intra-operative blood transfusion, while 1 patient who had a conversion to open splenectomy was given 2 units of packed cells post-operatively. The average preoperative requirement of blood transfusion per patient was 11.98 units per year (range, 5-20 units), whereas the average postoperative requirement of blood transfusion per patient came down to 4.04 units per year (range, 0-9 units) (*P*< 0.05) in the follow-up period of 6 months to 3 years. The mean splenic weight was 1038 gm (range, 284-1649 gm). There was no mortality in the current series; and except for excessive bleeding in 1 patient, no other major complication occurred intra-operatively. Postoperative complications included excessive vomiting in 4 (8%) patients; port site infection in 2 (4%) patients, which was controlled with local dressings and antibiotics. Four (8%) patients developed mild chest infection. One (2%) patient had prolonged drain output (tested negative for amylase) for 8 days, which resolved spontaneously. The average postoperative hospital stay was 4.7 days (range, 2-11 days).

## DISCUSSION

Since the introduction of laparoscopic splenectomy in 1991, it has evolved as a standard treatment for benign diseases with normal-sized spleens.[11] The definitions of massive splenomegaly vary among several published series, but all agree that as the splenic size and weight increase, the laparoscopic approach for splenectomy becomes a more difficult procedure, takes more time to complete and is more likely to end in a converted splenectomy.[12,13] Splenic size of over 30 cm along the longitudinal axis of the organ is considered a relative contraindication for the laparoscopic approach.[10,14,15] Splenectomy in patients with massive spleen remains a challenge even with conventional open surgery, with greater mortality and morbidity rates.[16,17] The degree of surgical difficulty is increased during laparoscopic splenectomy because the massively enlarged organ reduces the available intra-abdominal space to work. The problem associated with enlarged spleen becomes more evident in patients with β thalassemia as these patients usually have massive splenomegaly, that too in a smaller abdomen. Apart from the physical limitations to laparoscopic dissection and manipulation, thalassemic patients with massively enlarged spleens also have large vascular pedicles with splenic veins that can range up to 2 cm in diameter. Hence these patients pose greater risk of haemorrhage.[18,19] These patients often have hepatomegaly as well, which further compromises the working space.

In spite of various difficulties because of large spleen, there are a couple of advantages in patients of thalassemia. First, haemosiderosis associated with β thalassemia makes the spleen less friable and easier to manipulate. Secondly, the enlarged spleen displaces the splenic hilum anteriorly and inferiorly, providing direct and easy access to the splenic vessels.

The advantages offered by laparoscopic splenectomy over open method are obvious. It is associated with reduced analgesic requirements, rapid resumption of regular diet, shorter hospital stay, improved cosmetic outcome and lower societal cost.[18]

Two approaches have been described for laparoscopic splenectomy: the “anterolateral hanging spleen” technique described by Delaitre and Maignein[20] and the “posterolateral detached spleen” technique introduced by Park *et al*.[21] in 1992. We followed the anterior approach, starting the dissection at the lower pole. Early control of splenic artery was practiced using double ligatures with silk sutures. The splenic vein ligation was deferred till the remaining dissection was done except the division of splenophrenic ligament. This caused reduction in the size of the spleen up to 15%-20%, thereby increasing the working space and helping in the manipulation of the organ. It also caused reduction in the size of the splenic vein and its tributaries, thereby facilitating the dissection and reducing the risk of haemorrhage. The return of splenic blood into the systemic circulation was obviously in the interest of the already anaemic patients.

The major complication during laparoscopic splenectomy is haemorrhage, and it is the single most important reason for conversion to open procedure. Conversion rates reported in various series range from 6% to 36%.[7,8,22,23] Higher conversion rates were reported from studies which included higher percentage of patients with thalassemia having massive splenomegaly. The conversion rate in the present study was 2%. This was due to better case selection, surgical expertise and better instrumentation.

This decade has witnessed the evolution of hand-assisted laparoscopic splenectomy (HALS), as an alternative to laparoscopic splenectomy for patients with huge splenomegaly. Hand-assisted laparoscopic splenectomy offers various advantages, like better tactile sensation, easier exposure and manipulation of tissues, more effective retraction of tissues and organs, ease in achieving precise haemostasis and assistance in extracting the specimen through the accessory incision without morcellation.[11,24] HALS was not practiced in the current series. Most (52%) of the patients were ≤ 14 years of age with huge splenomegaly and hepatomegaly. The authors believe that a 7-cm incision used in HALS for the surgeon’s non-dominant hand was not only difficult due to hepatomegaly in a small abdomen but it would also have compromised the cosmetic value of laparoscopy. The 7-8–cm muscles-splitting pfannenstiel incision used for specimen retrieval was less morbid and cosmetically better than the same-sized incision used in HALS. It is also better than the left lower abdominal incision described in few reports for specimen extraction.[11]

Preoperative embolisation of splenic artery has been advocated to reduce operative time and blood loss during surgery of large spleens. However, due to serious complications, including severe pain, peri-splenitis and splenic abscess, this procedure has gone into disrepute.[15] Furthermore, preoperative embolisation did not significantly lower the risk of bleeding or of peri-operative blood transfusion in laparoscopic splenectomy patients. We did not follow this practice of preoperative embolisation in our patients.

The intra-operative blood loss and the need for intra-operative blood transfusion during laparoscopic splenectomy has been reported to be significantly more as compared to open splenectomy.[7] This complication is the major cause of conversions in most series. The mean intra-operative blood loss during laparoscopic splenectomy in our series was 73.8 ml, and none of the 49 patients in whom the procedure was completed laparoscopically required intra-operative blood transfusions. Only 1 patient, who was converted to open surgery due to excessive bleeding and adhesions, required blood transfusion (conversion rate, 2%).

Various studies[25] have reported that operative time in laparoscopic splenectomy is more than that in open splenectomy. We could not compare our findings of laparoscopic surgery and open surgery because of the selection bias involved in selecting the cases for open surgery. The overall mean operating time in 49 patients of splenectomy completed laparoscopically was 131 minutes, which is significantly lower than that reported in various other studies.[6,7] This may be due to the large number of patients operated by the authors and the same surgical team working together in better collaboration. The time required for laparoscopic splenectomy using pfannenstiel incision for specimen removal (124 minutes) was significantly less than the time required when the specimen was removed piecemeal by enlarging the umbilical port. This has also contributed to the overall lower mean operating time in the current series.

The mean hospital stay was 4.7 days, which is comparable with other studies on patients of thalassemia undergoing laparoscopic splenectomy.[2,7] This is lower than the hospital stay of patients undergoing laparoscopic splenectomy for other indications.[14] This is attributed to the characteristic psychological strength and strong will of the patients suffering from β thalassemia.[2] Overall morbidity was 22% although none of the complications required specific interventions. This included port site infection, prolonged drain output, excessive vomiting and mild chest infection. Postoperative blood transfusion requirement was reduced significantly, from 11.98 units to 4.04 units per patient per year (*P*< 0.05), in a follow-up period of 6 months to 3 years. This is in accordance with a sustained fall in transfusion requirement after splenectomy in patients of β thalassemia as reported in literature,[26] although more long-term follow-up is required to arrive at a definite conclusion.

## CONCLUSION

The overall findings of the present study eliminate any doubts regarding the feasibility of laparoscopic splenectomy even in the difficult setting of massive splenomegaly in patients of β thalassemia. The use of pfannenstiel incision for specimen retrieval significantly reduces operating time as compared to piecemeal removal of the specimen by enlarging one of the ports. It also carries low morbidity and is cosmetically better. It appears that factors for improving results include proper case selection, surgical expertise and teamwork supported by advanced technical facilities. We believe that experience of the surgeon is an important factor in successful completion of this procedure, as it holds true for any other laparoscopic surgery, indicating a relatively steep learning curve.
